# Body Mass Index Is Associated with Physical Performance in Suburb-Dwelling Older Chinese: A Cross-Sectional Study

**DOI:** 10.1371/journal.pone.0119914

**Published:** 2015-03-16

**Authors:** Suxing Shen, Jing Li, Qi Guo, Wen Zhang, Xiuyang Wang, Liyuan Fu, Linke Li, Yufang An, Weixi Liu, Hongyun Li, Tao Huang, Zedan Zhang, Kaijun Niu

**Affiliations:** 1 Department of Rehabilitation Medicine, Cardiovascular Clinical College of Tianjin Medical University, TEDA International Cardiovascular Hospital, Tianjin, China; 2 Department of Rehabilitation and Sports Medicine, Tianjin Medical University, Tianjin, China; 3 Key Laboratory of Hormones and Development (Ministry of Health), Metabolic Disease Hospital & Tianjin Institute of Endocrinology, Tianjin Medical University, Tianjin, China; 4 Nutritional Epidemiology Institute, Tianjin Medical University, Tianjin, China; 5 School of Public Health, Tianjin Medical University, Tianjin, China; Institute for Nutritional Sciences, CHINA

## Abstract

**Background:**

Physical performance is reported to have various beneficial effects on human health, especially in older individuals. Although such effects are associated with body mass index (BMI), the relationship between BMI and physical performance has not been clarified.

**Design:**

We conducted a cross-sectional study of 966 suburb-dwelling Tianjin individuals aged ≥ 60 years (average age 67.5±6.02, men 435, women 531). Mobility, balance, and muscle strength were assessed by walking speed, timed up-and-go test (TUGT), and grip strength, respectively. The subjects were categorized into three groups based on BMI (kg/m^2^) as follows: normal weight, 18.5 ≤ BMI ≤ 23.9; overweight, 24.0 ≤ BMI ≤ 27.9; and obese, BMI ≥ 28.0.

**Result:**

After adjusting for all other variables, relative grip strength decreased when BMI increased in both men and women (*P* for trend <0.001 and <0.001, respectively). BMI may be negatively associated with TUGT performance in the women only. There was no apparent association between walking speed and BMI in either sex, but after adjusting for age, walking speed was faster when BMI increased in women (*P* for trend= 0.0162).

**Conclusion:**

This study suggests that in older individuals, higher BMI is associated with poor muscle strength in both sexes.

## Introduction

In China, more than 14.3% of the population are aged 60 years or older [1]. It is well known that the aging process is characterized by gradual declines in muscle mass and strength, which ultimately lead to diminished physical performance [2], including high risks of falls and subsequent fractures, loss of independence, and increased morbidity and mortality rates [3–5]. An estimated 33 million Chinese older than 60 years have such limitations [6]; as such, attention should be given to the health of older individuals. Some researchers have reported that body mass index (BMI) is closely related to the overall health of the elderly [7, 8].

Recently, many researchers have studied the association between BMI and health. Besides, physical performance and health are also important issues. However, the relationship between BMI and physical performance in older individuals is still uncertain. Physical performance can be assessed by muscle strength, balance ability, and mobility. Several studies have reported that higher BMI is associated with reduced levels of physical performance. For example, in some studies, older adults with BMI (kg/m^2^) in the normal (18.5≤ BMI ≤22.9) or overweight range (23.0≤ BMI ≤24.9) had better walking speed than those with BMI in the obese (BMI ≥25.0) or severely obese range (BMI ≥30) [8, 9]. Other research studies have shown that overweight was not related to walking speed or timed up-and-go test (TUGT) performance [10, 11]. The relationship between BMI and grip strength (a marker of muscle strength) is inconsistent in older individuals; some studies have shown that higher BMI was associated with stronger grip strength [12, 13], whereas other results were inconsistent [8, 14].

The relationship between BMI and physical performance has demonstrated different results in different people [2, 8, 15, 16]. In China, more than 70% of the elderly live in suburban areas. Older individuals have a relatively low level of medical treatment and poor health [17], and particular attention should be given to those living in the suburbs [18]. Therefore, this study investigated suburb-dwelling individuals aged ≥ 60 years in Tianjin, China. The purpose of this study was to examine the association between BMI and physical performance in these older adults.

## Methods

### Study participants

For individuals aged 60 years or older (men 68.6±6.67, women 66.5±5.25), a free annual national physical examination is available. Based on this project, we made available a physical activity test at the township central hospital of Chadian, in the suburban area of Tianjin, China, from March to April 2013. In short, the exclusion criteria were as follows: (1) age < 60 years, (2) serious arthropathic deformation of the knee joint causing severe mobility impairment or localized loss of strength, (3) serious illness interfering with the conduct of the study or interpretation of the results, (4) current use of androgens or antiandrogens, (5) visual disorder with no appropriate clinical correction with corrective lenses at the time of the tests, and (6) refusal to participate in this study. Finally, a total of 966 older individuals (men 435, women 531) were recruited from the annual health examination. The study was approved by the ethics committee of Tianjin Medical University. Written informed consent was obtained from all the participants.

The examination included a questionnaire, physical performance tests, and grip strength measurement, which were conducted by postgraduate students in the health field who received special training for testing, refinement, and calibration of interviews. Subjects were categorized into different groups based on BMI, separately for men and women, to represent normal weight (18.5–23.9 kg/m^2^), overweight (24–27.9 kg/m^2^), and obese (≥28 kg/m^2^) using the criteria established by the Working Group on Obesity in China [19].

### Biochemical measurements

Blood samples were collected from peripheral veins of all the participants in the morning after a fasting period of 10 to 12 hours. Blood samples were collected in potassium EDTA tubes (Vacationer; BD, Oxford, United Kingdom) and immediately placed on ice. The samples were centrifuged for 15 minutes at 3000 rpm (AU5400; Olympus, Tokyo, Japan), and plasma was assayed for lipid profile, including total cholesterol (TC), triglyceride (TG), and fasting blood glucose (GLU) levels, using an automated analyzer.

### Questionnaire and anthropometric measurements

All the participants were invited to a face-to-face interview to answer a standardized questionnaire until they completed the medical examination. The questionnaire included data on age, education, profession, and disease history. The list of diseases, which were reported by the individuals, included diabetes, hypertension, hyperuricemia, stroke, coronary heart disease, arthritis and cancer. Information on smoking (“never,” “former,” and “current smoking”) and drinking (“never,” “former,” and “current drinking”) statuses also was obtained from the questionnaire. Smoking status included former smoking and current smoking; former smokers were those who had regularly smoked in the past, but had not smoked for at least half a year, and current smokers were those who smoked at least 1 cigarette per day lasting for at least 1 year. Information was obtained on the amount and type of alcohol that was consumed during the previous years, and alcohol drinking was defined as the consumption of at least 30g of alcohol per week for 1 year or more. Physical activity level was measured by the average number of hours per day spent in leisure, household, and occupational physical activities over the previous 7-day period. Physical activity was assessed by the short form of the International Physical Activity Questionnaire (IPAQ) in Chinese [20]. Responses were converted to metabolic equivalent task (MET) minutes per week [21] according to the IPAQ scoring protocol as follows: total minutes over the last 7 days spent on vigorous activity, moderate-intensity activity, and walking were multiplied by 8.0, 4.0, and 3.3, respectively, to create a MET score for each activity level. MET scores across the three subcomponents were summed to indicate overall physical activity [21]. Anthropometric measurements were conducted by well-trained examiners, with individuals wearing light clothing and no shoes. Height was measured to the nearest 0.1 cm using a portable stadiometer. Weight was measured to the nearest 0.1 kg using a calibrated scale, with the participant in the upright position. Weight and height were used to calculate BMI (kg/m^2^).

### Physical performance tests

To measure walking speed, two photocells connected to a recording chronometer were placed at the beginning and end of a 4-m course established at the site clinic. Participants were instructed to stand with both feet touching the starting line and to begin walking at their usual pace after a verbal command. The time between activation of the first and second photocells was recorded. The average time of two trials was used to compute walking speed. The participants were allowed to use a gait-assistance device [22]. The TUGT has been used to assess risks of basic balance and fall in older adults. The TUGT was administered by the researchers. The participants could wear their usual footwear and use a gait-assistance device, if needed. No physical assistance was given throughout the test. After receiving instructions to walk at their habitual gait speed, the participants had to sit on a standardized chair with the arms and trunk supported. After a verbal command to begin, the evaluator initiated the chronometer as the participant stood up, walked 3 m in a straight line, turned 180°, walked back to the chair, and sat down again. The clock was stopped only when the participant was seated in the chair with the arms and back supported. The time needed to perform the task was recorded in seconds [23].

### Grip strength measurement

Grip strength was measured using a hand dynamometer (Lafayette Instrument, TKK 5401; Takei Scientific Instruments Co., Ltd, Niigata, Japan). The test was conducted using the arm that was considered by the subject to be stronger. The participant held the hand dynamometer with the arm stretched out to the side of the body at a 45° angle and squeezed the grip as hard as possible while exhaling. Each subject made two attempts, with a 1-minute interval, and the maximum value (kg) was used for this study. Participants were encouraged to apply their maximum strength [14]. Grip strength relative to body weight (kg·kg^−1^) also was calculated because of involvement of body weight in the maximal performance of muscle strength [24, 25].

### Statistical analysis

Characteristics of the participants were reported as means (with 95% confidence intervals) or as percentages for anthropometrics, physical performance measures, and covariates (age, physical activity level, chronic diseases, and drinking and smoking statuses). Physical performance measures were used as dependent variables, while BMI categories were used as independent variables. Differences in variables between the BMI categories were examined using the analysis of variance for continuous variables or analysis of covariance (ANCOVA) for variables of proportion. For model 1, ANCOVA was used to examine relationships between BMI and physical performance, with adjustment for age. For model 2, the age variable was used, in addition to occupation, educational level, chronic diseases (diabetes, hypertension, hyperuricemia, stroke, coronary heart disease, arthritis and cancer), smoking and drinking status, and IPAQ score. The final ANCOVA (SAS glm proc) was performed with the forced entry of all factors considered to be potential covariates. Bonferroni-corrected P values were used for comparisons between the BMI groups. All the P values for linear trends were calculated by using the categories of BMI (18.5–23.9 kg/m2: 1; 24–27.9 kg/m2: 2; ≥28 kg/m2: 3). The association between the BMI and all the confounders, and their effect on physical performance were tested through the addition of cross-product terms to the regression model. Linear regression was used for calculating the coefficients in the regression model. A difference was defined to be significant at *P* < 0.05. All statistical analyses were performed using SAS version 9.2.

## Results

The baseline data of the participants according to BMI are shown separately for men and women in Table 1. The mean ages of the men and women were 68.6 ± 6.67 years and 66.5 ± 5.25 years, respectively. Occupation and educational level showed no significant differences between the BMI groups. Alcohol consumption showed no significant difference between the BMI groups (except for occasional drinking in men), as well as smoking status (except for former smoking in men). However, the percentages of female smokers and former drinkers in the normal weight group were higher than those in the other groups. History of hypertension was seen mostly in obese men and women. The percentage of female hyperuricemia in the obesity group was higher than the other two groups. With regard to physical activity level, the men in the overweight group had a significantly higher IPAQ score compared with those in the normal weight and obese groups. Meanwhile, IPAQ score was lowest for the overweight women among the BMI groups. In both the men and women, the TC, TG, and GLU levels showed no significant differences between the BMI groups. For men, the obese group had the worst walking speed and TUGT performance. In the women, there was no significant difference in walking speed between the normal weight and overweight or obese subjects; however, the mean value significantly increased with the increase in BMI. Among the other BMI groups, the normal weight group had the greatest relative grip strength.

**Table 1:** Subject characteristics according to categories of BMI.

	Male				Female			
Variables	Normal weight (BMI, 18.5–23.9)	Over weight (BMI, 24.0–27.9)	Obesity (BMI≥28.0)	P value	Normal weight (BMI, 18.5–23.9)	Over weight (BMI, 24.0–27.9)	Obesity (BMI≥28.0)	P value
**N**	153	208	74		170	231	130	
**Age (y)**	69.35(68.22,70.49)	68.53 (67.64,69.43)	67.23 (65.85,68.60)*	0.023	67.71(66.80,68.42)	65.97 (65.31,66.64)*	66.09 (65.21,66.98)*	0.005
**Weight (kg)**	64.01(63.05,64.96)	74.92 (74.07,75.78)*	85.31 (83.32,87.29)* †	<0.001	53.28(52.38,54.18)	64.87 (64.09,65.65)*	75.74 (74.25,77.24)* †	<0.001
**Height (cm)**	170.5(169.5,171.4)	169.7(168.8,170.5)	169.4(167.8,171.0)	0.376	156.2(155.2,157.2)	157.9 (157.1,158.7)*	157.5(156.4,158.6)	0.028
**BMI (kg/m^2^)**	22.00(21.78,22.23)	26.00(25.85,26.14)*	29.67(29.26,30.08)* †	<0.001	21.79(21.56,22.02)	25.97(25.82,26.12)*	30.45(30.09,30.82)* †	<0.001
**Famer (%)**	85.0	82.1	77.0	0.340	92.2	93.4	93.0	0.898
**Education (%)**								
**Primary or below**	77.1	74.5	81.1	0.509	85.9	89.2	85.5	0.593
**Secondary or above**	22.9	25.5	18.9	0.509	14.1	10.8	11.5	0.593
**Drinking status (%)**								
**Current**	36.6	29.1	28.4	0.256	1.2	1.3	1.5	0.967
**Occasional**	19.0	25.1	35.1	0.029	4.8	7.6	5.4	0.472
**Former**	19.0	25.6	18.9	0.250	3.0	0	0	0.005
**Never**	25.5	20.2	17.6	0.315	91.1	91.1	93.1	0.775
**Smoking status (%)**								
**Never**	33.3	30.9	31.1	0.877	45.2	63.6	72.1	<0.001
**Former**	20.9	33.3	29.7	0.035	11.3	12.3	9.3	0.693
**Current**	45.8	35.8	39.2	0.162	43.5	24.1	18.6	<0.001
**Hypertension (%)**	23.5	41.8	51.4	<0.001	39.4	52.4	60.8	0.001
**Diabetes (%)**	3.9	4.3	9.5	0.163	8.8	14.3	16.2	0.128
**Cancer (%)**	0.7	0.5	0	0.791	3.5	2.2	1.5	0.506
**Coronary heartdisease (%)**	15.0	17.3	16.2	0.846	27.6	31.6	35.4	0.354
**Stroke (%)**	6.5	9.6	6.8	0.514	2.9	7.8	8.5	0.080
**Arthritis (%)**	19.0	25.5	25.7	0.300	30.6	30.3	36.9	0.385
**Hyperuricemia (%)**	0.6	1.2	1.0	0.825	1.8	1.6	3.7†	0.406
**IPAQ scores**	3860 (3075,464)	5087(4195,5979)	4775 (3373,6177)†	0.146	4216(3461,4971)	2869(2423,3316)*	3746(2801,42691)	0.011
**TC (mmol/l)**	4.65(4.36,4.94)	5.10(4.18,6.01)	4.89(4.64,5.14)	0.676	5.07(4.93,5.22)	5.31(5.10,5.62)	5.84(4.62,7.06)	0.227
**TG (mmol/l)**	2.53(0.55,4.52)	1.60(1.45,1.74)	2.35(1.46,3.24)	0.484	2.45 (0.55,4.35)	4.22(0.93,7.52)	1.86(1.71,2.02)	0.435
**GLU (mmol/l)**	6.03(5.12,6.94)	5.86(5.54,6.18)	7.53(4.82,10.24)†	0.119	5.79(5.32,6.26)	6.28(5.56,6.99)	6.05(5.61,6.49)	0.526
**Grip /weight (kg/kg)**	0.52(0.51,0.54)	0.46(0.45,0.47)	0.44(0.42,0.46)*	0.002	0.38(0.37,0.39)	0.33(0.32,0.34)*	0.28(0.27,0.29)* †	0.014
**Walking speed (m/s)**	1.01(0.92,1.11)	0.97(0.93,1.00)*	0.96 (0.91,1.01)	0.543	1.04(1.00,1.07)	1.05(1.02,1.08)	1.08(1.03,1.12)	0.353
**TUGT (s)**	11.00(10.06,11.95)	10.50(10.10,10.91)	10.39(9.68,11.11)	0.461	10.72(10.33,11.10)	10.97(10.56,11.38)	11.14(10.61,11.69)	0.454

1 BMI, body mass index; IPAQ, International Physical Activity Questionnaire; TC, total cholesterol; TG, total triglycerides; GLU, fasting blood glucose.

2 Obtained by using ANOVA for continuous variables and chi-square for variables of proportion.

3 Mean; 95% CI in parentheses (all such values).

4 * p < 0.05 versus Normal weight; † p < 0.05 versus Over weight.

Adjusted associations between BMI and physical performance are shown in Table 2. For both the men and women, relative grip strength decreased across the BMI categories (*P* for trend < 0.0001 and < 0.0001, respectively). After adjusting for age, the significant negative relationship between BMI and relative grip strength still existed (*P* for trend < 0.0001 and < 0.0001, respectively). For men, when we compared to normal weight, the coefficients of overweight and obesity was -0.342 and -0.369, respectively (*P* < 0.0001). For women the coefficients of overweight and obesity was -0.325 and -0.541, respectively. They indicating that smaller relative grip strength were associated with bigger BMI in both men and women. The association between relative grip strength and the other confounders in the final models were also statistically significant. Both men and women, the coefficients of overweight and obesity was still singnificant (*P* < 0.0001). No association between BMI and TUGT performance was observed among the men (*P* for trend = 0.8314), even after adjusting for potential confounders. For the women, when not adjusting for potential confounders, the normal weight group had the shortest time, but no significant association between BMI and TUGT performance was observed. However, in model 1, after adjusting for age, a significant association appeared (*P* for trend = 0.0102). When we compared to normal weight, the coefficients of overweight and obesity was 0.111 and 0.118, respectively (*P* < 0.05). In model 2, after adjusting for confounders, the association remained significant (*P* for trend = 0.0325). But the coefficient was no significant. Although there was no significant among the groups, it was also exited the trend, the TUGT may be poorer with increasing the BMI. In the men, BMI was not associated with walking speed, even after adjusting for age, educational level, diabetes, hypertension, hyperuricemia, stroke, coronary heart disease, arthritis, cancer, smoking and drinking statuses, IPAQ score, and profession. In the women, after adjusting for age, BMI was associated with walking speed. The other confounders are independent of the walking speed.

**Table 2:** Adjusted the relationship between BMI and physical performance.

	BMI							*P* for trend^1^
Physical performance	Normal weight (BMI, 18.5–23.9)	Over weight (BMI, 24.0–27.9)	*β*	P value	Obesity (BMI≥28.0)	*β*	P value	
**Grip/weight (kg/kg)**								
No. of males	153	208			74			
Crude	0.51(0.49, 0.53)	0.45(0.44, 0.46)*	-0.320*	<0.001	0.43(0.40, 0.45)* ^4^	-0.319*	<0.001	<0.001
Age-adjusted^2^	0.52(0.50, 0.53)	0.45(0.44, 0.46)*	-0.342*	<0.001	0.42(0.40, 0.44)* ^†^	-0.369*	<0.001	<0.001
Multiple-adjusted^3^	0.60(0.49, 0.73)	0.52(0.43, 0.63)*	-0.336*	<0.001	0.49(0.40, 0.59)* ^†^	-0.360*	<0.001	<0.001
No. of females	170	231			130			
Crude	0.37(0.36, 0.38)	0.32(0.31, 0.33)*	-0.272*	<0.001	0.27(0.26, 0.28)* ^†^	-0.497*	<0.001	<0.001
Age-adjusted^2^	0.38(0.36, 0.39)	0.32(0.31, 0.33)*	-0.325*	<0.001	0.27(0.26, 0.28)* ^†^	-0.541*	<0.001	<0.001
Multiple-adjusted^3^	0.40(0.33, 0.48)	0.34(0.29, 0.41)*	-0.292*	<0.001	0.29(0.24, 0.35)* ^†^	-0.496*	<0.001	<0.001
**TUGT(s)**								
No. of males	153	208			74			
Crude	10.42(10.00,10.86)	10.29(9.93,10.66)	-0.056	0.299	10.03(9.45,10.64)	-0.054	0.317	0.292
Age-adjusted^2^	10.29(9.91,10.68)	10.30(9.97,10.63)	-0.037	0.463	10.27(9.73,10.83)	-0.012	0.812	0.960
Multiple-adjusted^3^	9.20(7.31,11.59)	9.31(7.43,11.65)	-0.050	0.335	9.27(7.34,11.70)	-0.026	0.618	0.831
No. of females	170	231			130			
Crude	10.46(10.11,10.83)	10.64(10.33,10.96)	0.045	0.380	10.81(10.40,11.25)	0.063	0.217	0.213
Age-adjusted^2^	10.25(9.93,10.57)	10.75(10.47,11.04)	0.111*	0.018	10.90(10.53,11.30)*	0.118*	0.012	0.010
Multiple-adjusted^3^	9.01(7.63,10.64)	9.41(7.96,11.13)	0.073	0.112	9.50(8.02,11.26)	0.069	0.138	0.033
**Walking speed(m/s)**								
No. of males	153	208			74			
Crude	0.95(0.92,0.99)	0.95(0.92,0.99)	-0.049	0.358	0.94(0.89,0.99)	-0.049	0.366	0.682
Age-adjusted^2^	0.94(0.91,0.98)	0.95(0.93,0.98)	-0.033	0.522	0.96(0.91,1.01)	-0.012	0.820	0.562
Multiple-adjusted^3^	0.86(0.69,1.07)	0.88(0.71,1.09)	-0.048	0.361	0.88(0.70,1.10)	-0.037	0.492	0.497
No. of females	170	231			130			
Crude	1.02(0.99,1.05)	1.02(1.00,1.05)	0.019	0.701	1.05(1.01,1.09)	0.073	0.150	0.203
Age-adjusted^2^	1.00(0.97,1.03)	1.03(1.01,1.06)	0.081	0.087	1.06(1.02,1.09)*	0.125*	0.009	0.016
Multiple-adjusted^3^	0.90(0.77,1.05)	0.92(0.79,1.08)	0.048	0.311	0.94(0.81,1.10)	0.078	0.102	0.058

^1^ Obtained by using ANCOVA;

^2^ Adjusted for age;

^3^ Additionally adjusted for educational level, profession; diabetes, hypertension, hyperuricemia, stroke, coronary heart disease, arthritis and cancer, history of smoking and drinking habits; International Physical Activity Questionnaire (IPAQ).

^4^ Mean; 95% CI in parentheses (all such values).

^5^ *p < 0.05 versus Normal weight;

†p < 0.05 versus Over weight.

## Discussion

The objective of this study was to examine the association between BMI and physical performance in older individuals. This study found that for the men, relative grip strength in the overweight and obese groups lower 13.3% and 18.3%, respectively, compared with the normal weight group. For the women, relative grip strength in the overweight and obese groups lower 15% and 27.5%, respectively, compared with the normal weight group. Age may be an important factor affecting balance among women. In TUGT performance, the obese group took 5.4% longer than the normal weight group. However, walking speed did not differ significantly between the groups. To the best of our knowledge, the present study is the first to examine the association between BMI and physical performance in suburb-dwelling older individuals in China.

In the present study, relative grip strength decreased significantly as BMI increased in both sexes. Even after adjusting for age and other confounders, the obese group had weaker grip strength compared with the other groups. Similar to our findings, Gunther et al. found that among anthropometric data of 769 healthy adults aged between 20 and 95 years, grip strength was correlated best with BMI [13]. Inconsistent with our results, however, Shin et al. reported that overweight and obesity were related to poor handgrip in a study of 97 postmenopausal women [14]. This discrepancy is possibly related to the characteristics of our cohort. Our study population comprised of individuals older (67.5 ± 6.02 years) than the participants in the study of Shin et al. (56.0 ± 4.3 years). Bassey et al. reported that muscle strength declines annually by approximately 1%–1.5% between 50 and 60 years of age and by 3% after 60 years of age [26]. We believe the association between BMI and grip strength was negative in the present study because testosterone level, which has a critical role in mediating improved muscle mass and strength, progressively declines with age [27, 28]. Furthermore, because BMI is associated with plasma steroid hormone concentration [29], grip strength decreases as BMI increases. We found that BMI was an independent factor of muscle strength in our study population.

In the present study, for the men, we found no significant association between TUGT performance and BMI. However, for the women, after adjusting for age and other confounders, a significant trend was found; but there was no difference among the groups. So the time to complete the TUGT test may became longer as BMI increased. Similar to our findings, Rebecca et al. found that higher BMI was associated with poorer TUGT performance in a study of eight UK cohort studies [12]. In contrast with previous reports, Ferreira et al. reported that BMI was not associated with the balance test in a study of 355 Brazilian older adults aged ≥ 60 years [11]. Our result is different than that of the study by Ferreira et al.; the reason might be the different races [2]. Our study found that the association only appeared in the women; due to genetic, hormonal, and environmental differences, women tend to have a greater proportion of fat mass compared with men [30, 31], and excess fat deposition around the muscle fibers may interfere with function and reduce muscle quality [32]. Besides, in this study, obesity was associated with increased burden of chronic disease, which has been shown to negatively affect dynamic balance [33, 34]. In the present study, individuals with obesity were more likely to have hypertension, diabetes, or coronary heart disease compared with those who were normal-weight or overweight. The association between BMI and dynamic balance was partially explained by these factors; however, the data suggest that even after adjusting for many potential confounding factors, BMI may be independently related to dynamic balance only in the women. Consequently, the TUGT may became longer as BMI increased, and the obese women would had a higher risk of falling.

In our study, there was no sex-related difference in walking speed between the BMI groups. When we adjusted for age, an association appeared only in the women. When we controlled for the other influential confounders, the difference disappeared in both the men and women. Sergi et al. also showed that the association between walking speed and BMI in older adults was dependent on sex, age, and type of test performed, only in women was low BMI associated with higher probability of overall motor performance impairment [10]. In contrast with our results, in a study by Wool et al. involving Hong Kong residents aged ≥ 65 years, those with BMI ≥ 30 kg/m^2^ had the worst walking speed [8]. One study reported that rates of loss in balance and gait speed were both similar for men and women, with balance deteriorating at an earlier age than gait speed (60–70 years vs 70+ years) in both sexes [35]. While it is difficult to explain this finding, some studies have shown that socioeconomic inequalities and/or socioeconomic development and income inequality have a negative effect on health condition, including physical performance [36]. Our subjects lived in the suburbs, and most of them were farmers, whereas the Hong Kong residents lived in the city, and all of them were citizens, in addition to the fact that the economy of the city is better than that of the suburbs. A prospective study of Swedish adults aged ≥ 50 years reported no significant differences in timing (age) or rate (magnitude) of decline in grip strength, balance, or gait according to sex. Based on our results, we can draw the conclusion that BMI has no correlation with walking speed.

This study also has several limitations. First, the participants who were unable to complete the tests or who refused to perform them, and the participants who were not able to come or did not want to participate in the tests, can be understood as a bias in the analysis, thus our findings may be confounded by selection bias. Therefore, our results might not represent the general elderly population. Second, although we adjusted for a considerable number of confounding factors, we cannot exclude all the possibility confounders that may be affected the result, such as social economic or lifestyle factors, which correlate with BMI. Therefore, an intervention study is necessary for establishing a causal relationship between BMI and physical performance. Third, we use BMI as an indicator of adiposity is our another limitation, BMI was widely used to estimate the nutritional status in many articles. BMI does not distinguish between fat mass and muscle mass; nor does it allows for fat infiltration in muscle or sarcopenia, we although relatively consistent links have been established between BMI and physical disability [37, 38]. These findings suggest that this simple anthropometric measure is useful for screening older individuals with functional limitations. Forth, because this study was cross-sectional, we could not establish causal relationships. As such, a prospective study or trial should be undertaken to confirm the relationship between BMI and physical performance.

## Conclusion

In suburb-dwelling older individuals, BMI is associated with muscle strength in both sexes. Among the elderly, individuals with normal weights may have optimal grip strength. Age may be an important factor of mobility and balance in women. However, mobility and balance are independent of BMI. Decline in muscle strength may be earlier than declines in balance and mobility. Particular attention should be given to suburb-dwelling elderly. Further research, such as longitudinal studies, is required in this area to gain better understanding of the impact of focusing on the care of older individuals with disabilities.
